# Presentation Attack Detection for Iris Recognition System Using NIR Camera Sensor

**DOI:** 10.3390/s18051315

**Published:** 2018-04-24

**Authors:** Dat Tien Nguyen, Na Rae Baek, Tuyen Danh Pham, Kang Ryoung Park

**Affiliations:** Division of Electronics and Electrical Engineering, Dongguk University, 30 Pildong-ro 1-gil, Jung-gu, Seoul 100-715, Korea; nguyentiendat@dongguk.edu (D.T.N.); naris27@dongguk.edu (N.R.B.); phamdanhtuyen@gmail.com (T.D.P.)

**Keywords:** iris recognition, presentation attack detection, convolutional neural network, support vector machines

## Abstract

Among biometric recognition systems such as fingerprint, finger-vein, or face, the iris recognition system has proven to be effective for achieving a high recognition accuracy and security level. However, several recent studies have indicated that an iris recognition system can be fooled by using presentation attack images that are recaptured using high-quality printed images or by contact lenses with printed iris patterns. As a result, this potential threat can reduce the security level of an iris recognition system. In this study, we propose a new presentation attack detection (PAD) method for an iris recognition system (iPAD) using a near infrared light (NIR) camera image. To detect presentation attack images, we first localized the iris region of the input iris image using circular edge detection (CED). Based on the result of iris localization, we extracted the image features using deep learning-based and handcrafted-based methods. The input iris images were then classified into real and presentation attack categories using support vector machines (SVM). Through extensive experiments with two public datasets, we show that our proposed method effectively solves the iris recognition presentation attack detection problem and produces detection accuracy superior to previous studies.

## 1. Introduction

Over recent decades, biometric technology has gained much attention and is widely used in various applications to enhance user convenience and the security level of recognition systems compared to traditional recognition methods [1,2,3,4,5,6,7,8,9]. However, researchers have recently indicated that biometric recognition systems are vulnerable to attack by attackers presenting fake samples to data collecting systems [2,10,11,12,13,14,15,16]. Using appropriate artificial biometric features, an unauthorized person can be recognized as authorized by a biometric recognition system using either direct or indirect attack methods [16]. As a result, presentation attack detection methods are required to protect a biometric recognition system from attackers and enhance its security level.

Among the many biometric features, the iris pattern has been recently used for recognition because of its reliability and high security [3,9]. However, several studies have indicated that a fake iris pattern can be made by recapturing a real iris pattern or by printing an iris pattern on a contact lens to fool iris recognition systems. To address this problem, we propose a new presentation attack detection method for an iris recognition system by using hybrid image features and offer a classification method to overcome the limitations of previous research. Our proposed method is novel in five ways compared to previous research.

- First, this is the first approach to use a deep CNN model for iPAD to overcome the limitation of previous studies which adopted only shallow CNN networks. The trained CNN model can extract discriminative features for classifying real and presentation attack images because it is trained using a large amount of augmented training images.
- Second, since presentation attack images have special characteristics such as noise or discrete patterns of textures, we applied a multi-level local binary pattern (MLBP) method to extract these images features. The handcrafted image features can be seen as a complement to the deep features to enhance the classification result.
- Third, we combined the detection results based on MLBP and deep features to enhance the accuracy of the iPAD method. The combination was performed using feature level fusion and score level fusion. This is the first approach to combine handcrafted and deep features for iPAD.
- All previous research showed the performances of iPAD according to the individual iPAD dataset such as printed or contact lenses. However, we present the robustness of our method irrespective of the kinds of iPAD datasets through the evaluation with the fused datasets of printed and contact lenses.
- Finally, we made our trained models and algorithms for iPAD available to other researchers for comparison purposes [17].

## 2. Related Works

Previously, several methods have been proposed for detecting presentation attack images for iris recognition systems [18,19,20,21,22,23,24]. Generally, these studies can be classified into two groups, including iPAD methods based on expert-knowledge (handcrafted) image features and iPAD methods based on learning-based image features.

In the first group, authors mainly designed several feature extraction methods based on their expert knowledge of the problem. With the extracted image features, they performed classification methods such as support vector machines to detect real and presentation attack images [18,19,20]. One example of the first group for the iPAD method is the work by Gragnaniello et al. [18]. In this work, several local descriptors were used to detect iris images. Local descriptors such as the local binary pattern (LBP) and its variants, local phase quantization (LPQ), binarized statistical image features (BSIF), and shift-invariant descriptors (SID) were proven to be effective for detecting presentation attack images. However, as shown in their experimental results, the detection accuracy varied according to the kind of feature extraction methods and working datasets and reduced the reliability of the detection system. The BSIF feature extraction method was successfully used in a study by Doyle et al. [19] for detecting the textured contact lenses in an iris recognition system. One important result obtained from this study was that the accurate segmentation of the iris region is not required to obtain accurate detection results. In a study by Komogortsev et al. [21], the eye movement information was used for iris liveness detection. However, eye movements can be simulated by imposters who have expert-knowledge of the problem. Instead of using a gray-textured image, Raja et al. [22] used the information from different color channels to detect a presentation attack ocular image. As indicated from these studies, the handcrafted image features were effective for detecting presentation attack iris images.

In the second group, authors leave the details of feature extraction and classification behind the scenes by applying a learning-based method on a large amount of training data to train a detection model. For example, Silva et al. [23] used a convolutional neural network (CNN) called spoofnet to detect textured cosmetic contact lenses. Experimental results using the Notre Dame Contact Lens (NDCL-2013) dataset showed that the CNN method produced state-of-the-art detection results. However, using the IIIT-Delhi dataset, the CNN method produced less than state-of-the-art results. In addition, the spoofnet used in this research was relatively shallow (two convolution layers and one fully connected layer). This problem can affect the detection accuracy. Similar to this research, Menotti et al. [24] used a CNN network by applying two optimization schemes including structure optimization and filter optimization. They validated the detection performance for various biometric features such as face, fingerprint, and iris. Their proposed method combining the architecture and filter optimizations worked well for the fingerprint benchmark. However, their face and iris benchmarks produced detection results just comparable with state-of-the-art results. Again, the CNN networks used in this research were relatively shallow with two convolution layers and one fully connected dense layer. The results of these studies demonstrate that a deep convolutional neural network is effective for detecting presentation attack images for biometric recognition systems. However, in addition to the scarceness of training data, the use of a shallow network architecture can be a limitation of these studies. In Table 1, we summarize previous studies by considering the detection methods with their strengths and weaknesses.

The rest of our paper is organized as follows. In Section 3, we present the main structure of our proposed iPAD method and a detailed description of the technique. In Section 4, we perform various experiments using two public datasets to evaluate the detection performance of our proposed iPAD method and compare our experimental results with those of previous research and discuss our results. Finally, we provide concluding remarks in Section 5.

## 3. Proposed PAD Method for Iris Recognition System

### 3.1. Overview of Proposed Method

**Figure 1:**
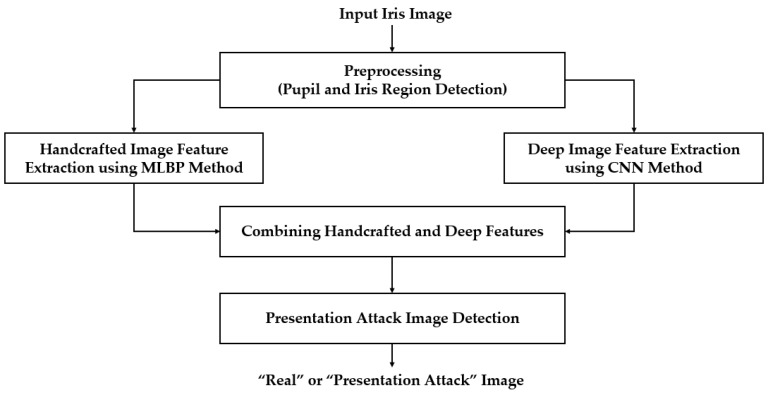
General flowchart of proposed iPAD method.

Figure 1 shows the overall flowchart of our proposed iPAD method. Similar to an iris recognition system, we first detected the iris region from the input iris image to localize the iris region. This step was necessary because the iris region can differentiate between a real and presentation attack iris image, while the other regions contain no or less discrimination information according to the attack method. Based on the detection result of this step, we extracted an iris region of interest and used this image to extract features for our proposed method. The detailed explanation of this step is given in Section 3.2.

We then extracted the image features in the localized region of interest produced by the preprocessing step. Our proposed method extracted the handcrafted features and deep features using the MLBP and a CNN method, respectively. The details of these image feature extraction methods are provided in Section 3.3 and Section 3.4, respectively. As a result, we obtained a feature vector for the MLBP method and for the CNN method. These two feature vectors were then combined using feature level fusion and score level fusion approaches. A detailed description of each fusion method is provided in Section 3.5. Finally, we used a SVM to classify the input image into real and presentation attack classes using the extracted image features.

### 3.2. Iris Region Detection Using Circular Edge Detection Method

**Figure 2:**
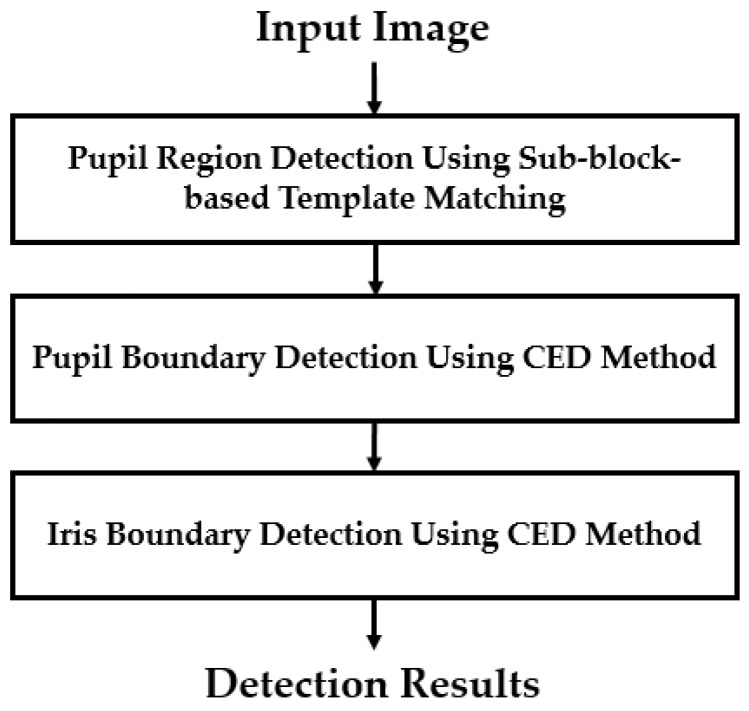
Flowchart of iris segmentation method in our study.

Since an iris recognition system uses the iris region to recognize individuals, attackers to this system attempt to create a presentation attack sample that is similar to that of the real image. Therefore, the iris region probably contains more discrimination information between real and presentation attack images than the sclera and skin regions in an iris image. Based on this observation, the first step in our proposed method was designed to detect the iris region in an input iris image. To efficiently detect the iris region, our proposed method used a sub-block-based template matching procedure to roughly detect the pupil region based on the characteristics of the iris image. Based on the result of pupil region detection, we continued to roughly localize the image region in which the iris region exists. Finally, we used the CED method to accurately detect the boundaries of the iris region as shown in Figure 2.

**Figure 3:**
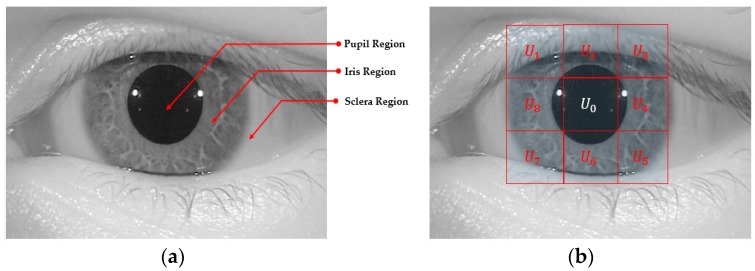
Block-based method for pupil region detection. (**a**) Pupil, iris, and sclera regions of eye image. (**b**) Example of 9 sub-blocks on pupil and iris regions.

**Figure 4:**
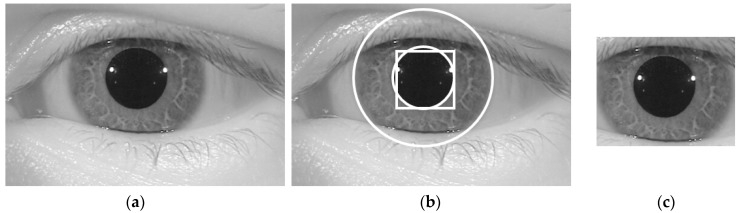
Example of detection results of pupil and iris boundary detection method: (**a**) input iris image; (**b**) detection results of sub-block-based pupil detection (rectangular box) and CED for pupil and iris region detection; and (**c**) final iris image to input iPAD system.

Inspired by the observation that the iris region of the human eye is displayed as a circular shape region in the iris image, the iris boundaries can be effectively detected by the CED method [25]. Although we can detect the iris boundaries using the CED method by searching the entire image, it incurs a long processing time because we must search the boundaries at various center positions and potential radius values. In addition, the effect of noise and abnormal texture can affect the detection result. To overcome this problem, our proposed method used a preprocessing method called the sub-block-based template matching method to detect the pupil region roughly first before detecting the iris boundaries using the CED method. Using NIR light, iris images are normally captured with a pupil region that is darker than other regions such as the iris sclera and skin regions. This characteristic is caused by the different absorption and reflection of NIR light in different regions of the human eye. Based on this characteristic, we used a sub-block-based template matching method to first localize the pupil region in a given iris image. The sub-block-based template matching was performed by measuring the difference in gray-levels of the sub-blocks that surround the pupil region with the center sub-block as shown in Figure 3. In this figure, at a center position (*x*, *y*) with block-size (s), we denote *U*_0*,x,y,s*_ as the average gray-level of the center sub-block and *U_i,x,y,s_* (*i* = 1, …, 8) as the average gray-levels of the surrounding sub-blocks. As a result, if the center sub-block contains the pupil region, its average gray level (*U*_0*,x,y,s*_) is much smaller than those of the surrounding sub-blocks (*U_i,x,y,s_*). Based on this observation, we detected the pupil region in a given iris image by using Equation (1) with the condition that *U*_0*,x,y,s*_ is smaller than *U_i,x,y,s_* (*i* = 1, …, 8). Furthermore, to speed up the processing of this step, the integral image was used to quickly calculate the average gray-level of the sub-blocks [26]. An example result of the pupil detection step is shown in Figure 4 with a rectangular bounding box.

(1) $$\underset{x,y,s}{\mathrm{argmax}}\left(\overset{8}{\underset{i=1}{\sum }}\left(U_{i,x,y,s}-U_{0,x,y,s}\right)\right)$$

We then accurately detected the iris boundaries based on the detection result of the pupil region using the CED method [25,26,27]. As shown in Figure 3 and Figure 4, the center of the iris and pupil regions are pixels inside the bounding-box of the pupil region. In addition, the radius of the pupil region is smaller than that of the iris region. Based on this observation, we used two circular edge detectors to find the boundaries of the pupil and iris regions. The pupil region normally appears as a complete circle. Therefore, we first used the complete circular edge detector shown in Equation (2) to detect the boundary of the pupil region. In this equation, *r* and (*x_c_*, *y_c_*) are the radius and center position of the pupil region. However, the iris region can be occluded by some additional regions such as the eyelid, eyelash, or eyebrow. As a result, the boundary of the iris region can be not continuous. To overcome this problem, we used the CED method in a limited circular range. As suggested by previous research [26], we used the circular range of −45° to +30° and +150° to +225° as shown in Equation (3). In this equation, *r’* and (*x’_c_*, *y’_c_*) are the radius and center position of the iris region. In Figure 4, we show an example of the result of our iris detection method.

(2) $$\underset{x_{c},y_{c},r}{\mathrm{argmax}}\left[\frac{\partial }{\partial r}\int_{0}^{2\pi }\frac{I\left(x_{c}+r\cos\theta ,y_{c}+rsin\theta \right)}{2\pi r}d\theta \right]$$

(3) $$\underset{x_{c}^{'},y_{c}^{'},r^{\prime }}{\mathrm{argmax}}\left[\frac{\partial }{\partial r}\left(\int_{-\frac{\pi }{4}}^{\frac{\pi }{6}}\frac{I\left(x_{c}^{'}+r^{\prime }cos\theta ,y_{c}^{'}+r^{\prime }sin\theta \right)}{5\pi r^{\prime }/12}d\theta +\int_{\frac{5\pi }{6}}^{\frac{5\pi }{4}}\frac{I\left(x_{c}^{'}+r^{\prime }cos\theta ,y_{c}^{'}+r^{\prime }sin\theta \right)}{5\pi r^{\prime }/12}d\theta \right)\right]$$

As shown in Figure 1, our proposed iPAD method uses CNN method for extracting deep image features. As we will show in next section, the CNN network requires the 3-channel input images. To make the input images for CNN network, we localized the iris region of interest (ROI) based on the detection results of pupil and iris detection method and made the final iris images for iPAD system by scaling the iris ROIs to the size of 224-by-224-by-3 images using bilinear interpolation method. Because the iris ROI is gray image, we duplicated it into the 3 channels, and obtained the 3-channel image. In Figure 4c, we showed an example of iris image that is used to input into iPAD system in our study.

### 3.3. Image Feature Extraction Based on MLBP Method

**Figure 5:**
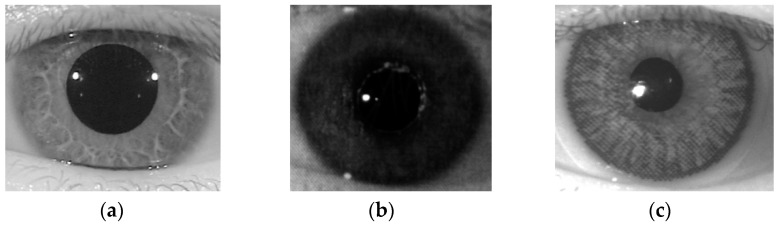
Example of NIR iris images: (**a**) real image; (**b**) presentation attack image obtained by recapturing a printed sample on paper; and (**c**) presentation attack image obtained by recapturing a contact lens.

In Figure 5, we show an example of one real and two presentation attack iris images according to two different attack methods using a printed image and a contact lens. As shown in this figure, while the real iris image contains very clear iris patterns and fine texture features, the presentation attack images contain dot noise and broken textures (Figure 5b,c) because of the effects of printed iris patterns on paper or on a contact lens. Based on this observation, our proposed method used the LBP method to extract the image features for the iPAD.

As indicated by previous studies, the LBP method is a very efficient image feature extraction method in image processing and computer vision research by providing illumination and rotation invariant characteristics to extracted image features [28,29,30]. Furthermore, the LBP descriptor describes well the micro-texture features such as blob, edge, corner, and flat regions. By definition, the LBP method encodes each center pixel of a given image by a sequence of *P* (bits) using *P* surrounding pixels of the center pixel with a radius of *R* as shown in Equation (4). The LBP operator works as an adaptive thresholding function and offers the illumination invariant to the image features extracted by the LBP method.

(4) $${\mathrm{LBP}}_{R,P}=\sum_{i=0}^{P-1}s\left(g_{i}-g_{c}\right)2^{i}\text{ where }s\left(x\right)=\{\begin{array}{c} 1\;if\;x\geq 0\\ 0\;if\;x\;<0 \end{array}$$

**Figure 6:**
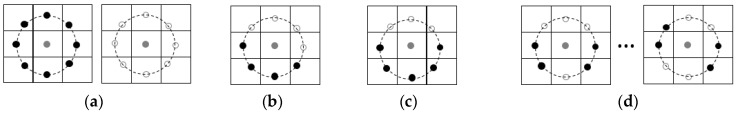
Example of LBP descriptors for representing micro-texture features: (**a**) flat/blob textures; (**b**) edge texture; (**c**) corner texture; and (**d**) complex noise-sensitive texture features.

To extract the image features for the iPAD, we classified the LBP descriptors of pixels in a given image into two categories of uniform and non-uniform patterns. By definition, the uniform patterns are patterns that have at most two bit-wise transitions from 0 to 1 or 1 to 0, and the non-uniform patterns are those that have more than two bit-wise transitions from 0 to 1 or 1 to 0. The reason for this classification is that the uniform patterns effectively describe various useful micro-texture features such as blob, corner, edge, or flat regions [28,29,30], while the non-uniform patterns are complex and normally caused by noise and non-uniform texture patterns. In Figure 6, we show an example of the ability of an LBP descriptor to represent several micro-texture features such as blob, corner, and edge. As we explained at the beginning of this section, the definition of the LBP method is suitable for discriminating between real and presentation attack images because the presentation attack iris images can contain dot noise and non-ideal image texture features.

As the final step, we constructed the image feature vector by accumulating the histogram of uniform and non-uniform patterns over the image. The histogram features effectively describe the characteristics of image texture because the histograms of uniform and non-uniform patterns statistically measure the distribution of micro-texture features over an iris image. Suppose that we used an LBP operator with radius *R* and number of surrounding pixels *P* to extract image features, the dimension of the extracted image features is given by Equation (5).

(5) $$DIM_{\mathrm{LBP}}=P\times \left(P-1\right)+3$$

As suggested from previous studies, our study accumulated the LBP features for an iris image by concatenating histogram features obtained from hyper-parameters such as radius (*R*) and number of representation pixels (number of surrounding pixels, *P*). The MLBP method was used to capture richer information from iris images than conventional LBP methods [30]. In our experiment, we used various values for radius (*R* in range from 1 to 3) and number of surrounding pixels (*P* of 8, 12, and 16) for MLBP feature extraction method. As a result, we extracted a 933-dimensional image feature vector for iPAD.

### 3.4. Image Feature Extraction Based on CNN Method

As shown in Figure 1, our proposed method used MLBP and CNN methods to extract image features for iPAD. While the MLBP is a hand-designed feature extraction method, the CNN method is a learning-based feature extraction method based on a learning procedure to learn a model that is applicable for feature extraction and classification. In literature, this method has been successfully used in various computer vision systems such as image classification [31,32,33,34], object detection [35,36], face recognition [37], gender recognition [38], and even the PAD problem [2,22,23]. As shown in these studies, the CNN method can produce state-of-the-art results compared to previous hand-designed methods. In the field of iris recognition, the CNN method has also successfully used and provided state-of-the-art recognition accuracy [39,40]. In the study by Gangwar et al. [39], two deep CNN networks named as DeepIrisNet-A (with 8 convolutional layers and 3 fully connected layers) and DeepIrisNet-B (with 5 conventional convolutional layers, 2 inception layers, and 3 fully connected layers) were used for iris recognition. The results of this study show that the CNN method is effective at not only enhancing the recognition accuracy but also robust to cross-sensor recognition. In a recent research conducted by Nguyen et al. [40], they used several pre-trained CNN models including AlexNet, VGGNet, InceptionNet, ResNet, and DenseNet to extract image features for iris recognition. Based on their experimental results, the CNN method outperformed the baseline iris recognition method although the CNN models were trained for a different task. Inspired by these previous studies, we used the CNN method to extract the deep features for iPAD.

**Figure 7:**
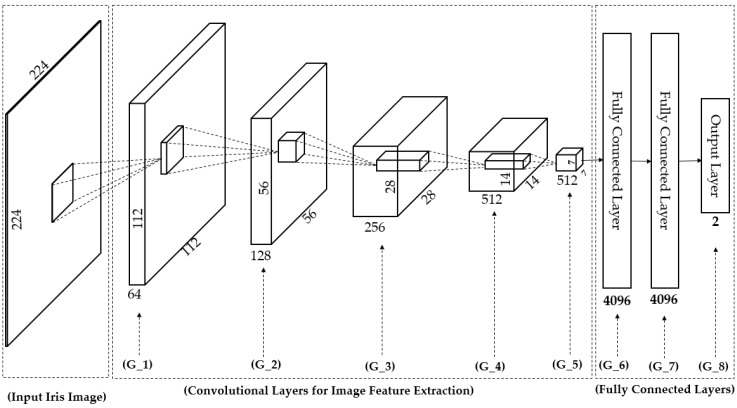
Visualization of convolutional neural network architecture in Table 2.

In Table 2, we provide a detailed description of the CNN network architecture in our study. The CNN network was based on the very deep network proposed by Simonyan et al. [32] called VGG Net-19. The network architecture is depicted in Figure 7. Generally, a CNN network consists of two main components of convolution layers and fully-connected layers [31,32]. The convolution layers are responsible for image manipulation to extract image features using an image filtering technique, and the fully-connected layers are used to classify the extracted image features into several categories of desired class labels. In addition to these two main components, a CNN model can contain several layers such as activation layers (using sigmoid, tanh, or rectified linear unit (ReLU) functions), pooling layers (max or average pooling), and SoftMax layers. As shown in Table 2 and Figure 7, our CNN network consisted of 19 weight layers (16 convolution layers and three fully-connected layers) followed by several ReLU and max pooling layers. In addition, the last fully-connected layer in our study contained only two neurons which stand for “real” and “presentation attack” classes instead of the 1000 neurons used in the original VGG Net-19 [32]. In this table, we grouped several convolution layers which have same parameters together as denoted as G_1, G_2… G_8 in Table 2 and Figure 7. For example, the G_0 group contains two convolutional layers which have same parameters of the number of filters (64 filters), filter size (3 × 3 pixels), stride (1 × 1 pixel) and padding (1 × 1 pixel). The output of convolutional layers is 512 feature maps of the size of 7 × 7 pixels taken at the end of the G_5 group. In total, we obtained 25,088 activation neurons after 16 convolutional layers. These output neurons are connected to 4096 neurons in the next fully connected layer of the G_6 group by fully interconnection based on weighted summation. For example, the value to the 1st one of 4096 neurons is calculated by *w*_1_ × *o*_1_ + *w*_2_ × *o*_2_ + … + *w*_25088_ × *o*_25088_ where *o*_1_, *o*_2_, … *o*_25088_ are the values from 25,088 activation neurons, and *w*_1_, *w*_2_, … *w*_25088_ are the weights for interconnection.

An optimal CNN model for a given problem can be obtained using a training procedure using a large amount of training data through which the filter’s coefficients and weights of fully connected layers are efficiently learned with respect to the ground-truth labels of images. However, the CNN method always faces the problem of over-fitting because the network contains a very large number of parameters (filter coefficients and weights of fully connected layers) and because of the small training dataset and/or poor network parameter initialization. To reduce the over-fitting problem of the CNN network, we applied the dropout method to the first two fully-connected layers with a dropout value of 0.5. In addition, we used a pre-trained model that was successfully trained using ImageNet dataset [32] to initialize the weights of our CNN model. With the initialized network, we re-trained the whole network parameters (training from scratch). This is different procedure form conventional transfer learning [41]. We used the stochastic gradient descent method with momentum to train the CNN models [31]. The detailed parameters of training process are given in Table 3. To extract the image features using the CNN method, we extracted the activations of the second fully-connected layers (G_7 in Figure 7) and used them as the extracted features of the input images. Although it is possible to use the other layers (convolution layers or fully-connected layers) for feature extraction, the use of the deeper layer contains more information than that of the shallower layers. As a result, we extracted a 4096-component feature vector for our proposed iPAD.

### 3.5. Image Feature Extraction and Detection Using SVM Method

Using the two feature extraction methods mentioned in Section 3.3 and Section 3.4 (i.e., MLBP and CNN), we extracted two corresponding feature vectors of *f*_1_ and *f*_2_ for the MLBP and CNN features, respectively. These two feature vectors can contain different information for our iPAD because they were extracted using two different methods. As the main contribution of our proposed method, the information from the two feature vectors was combined to enhance the detection accuracy of the iPAD system. As explained in Section 3.1, we used the feature level fusion and score level fusion approaches for this step.

**Figure 8:**
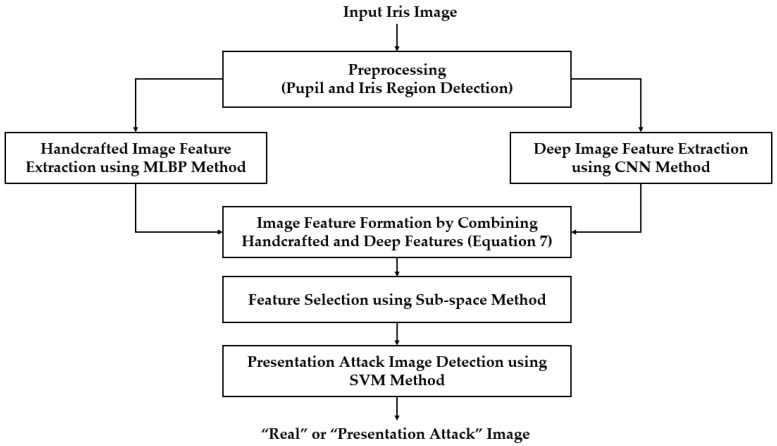
Flow chart of proposed iPAD method based on feature level fusion approach.

For the first fusion method, we combined the two vectors to form a new feature vector called the hybrid feature vector, to represent the input image. As a result, the flowchart of our proposed method in Figure 1 changed to that of Figure 8. For this purpose, we first normalized each feature vector to a zero-mean and unit standard deviation using the z-score normalization method shown in Equation (6) [28]. In this equation, *f_mean_* and σ are the mean and the standard deviation vector obtained by a training dataset, respectively. Using this equation, we normalized the extracted feature vectors *f*_1_ and *f*_2_ and obtained the two corresponding normalized feature vectors, $f_{1}^{norm}$ and $f_{2}^{norm}$. Finally, the hybrid feature *f_hybrid_* was formed by simply concatenating the two normalized feature vectors as shown in Equation (7).

(6) $$f^{norm}=\frac{f-f_{mean}}{\sigma }$$

(7) $$f_{hybrid}=\left[f_{1}^{norm},f_{2}^{norm}\right]$$

Although we can extract richer information to combat presentation attacks by using the hybrid feature vector rather than using only the MLBP or CNN feature vector, the iPAD system must process data in a higher dimensional space in later steps (classification step) than that of an individual feature vector. This problem increases the processing time for both the training and testing phases and the complexity of the classification model. To overcome this problem, we further reduced the dimension of the hybrid feature vector using a subspace method called principal component analysis (PCA). This well-known method reduces the dimension of data by constructing a low dimensional space in which the original data are well represented [28,30]. Originally, we extracted a 4096-dimensional feature vector using CNN-based method using the second fully-connected layer of CNN network in Table 2. For the MLBP feature, we extracted image feature using various values of LBP parameters (radius (*R*) from 1 to 3 and resolution (*P*) of 8, 12 and 16). Consequently, we extract a feature vector in 933-dimensional space. As a result, the hybrid feature vector is a 5029-dimensional vector. In our experiments, we used the PCA for obtaining the optimal dimension of features before using SVM method for classification. In details, we used the number of principal component of 512 which is much smaller than the dimension of original features. The use of this reduced number of feature dimension helps us to lessen the complexity of classifiers, processing time, and effects of noise. As the final step of this fusion approach, we classified the input image into real and presentation attack classes using extracted image features. For this purpose, we used an up-to-date classification method based on SVMs for the classification problem. Conventionally, the SVM method constructs a classifier using several data points called support vectors and uses it to classify new input features into classes by evaluating the sign of evaluation function in Equation (8). In this equation, *x_i_* and *y_i_* are the support vectors and its corresponding class label, *a_i_* and *b* are the parameters of the classifier, and $K(x,x_{i})$ is the SVM kernel function, a hyper-parameter of the SVM method [42]. These classifier parameters are trained using training data and saved to predict the class label of new input features. In our experiments, we used three different kinds of kernel functions, including the linear, radial basis function (RBF), and polynomial kernel functions as shown in Equations (9)‒(11) [42,43,44].

(8) $$f\left(x\right)=sign(\overset{k}{\underset{i=1}{\sum }}a_{i}y_{i}K\left(x,x_{i}\right)+b)$$

(9) $$\mathrm{Linear}\;\mathrm{kernel}:\;K\left(x_{i},x_{j}\right)=x_{i}{}^{T}x_{j}$$

(10) $$\mathrm{RBF}\;\mathrm{kernel}:\;K\left(x_{i},x_{j}\right)=e^{-\gamma \|x_{i}-x_{j}\|{}^{2}}$$

(11) $$\mathrm{Polynomial}\;\mathrm{kernel}:\;K\left(x_{i},x_{j}\right)={\left(\gamma x_{i}{}^{T}x_{j}+coef\right)}^{degree}$$

**Figure 9:**
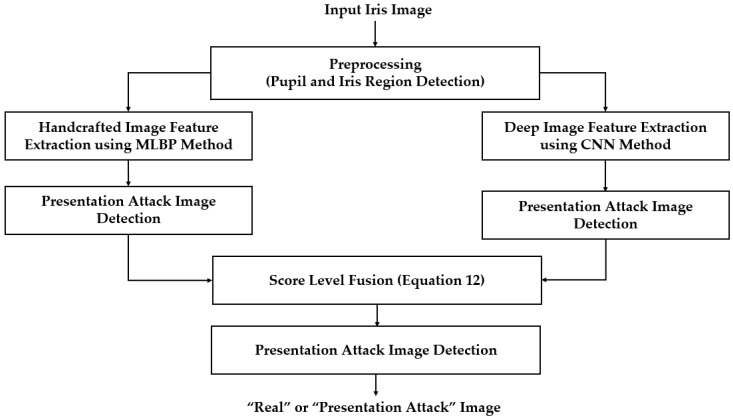
Flow chart of proposed iPAD method based on score level fusion approach.

Moreover, the combination of handcrafted and deep features can be done by another combination method called score level fusion [45]. For this combination method, the overall detection system in Figure 1 changed to that of Figure 9. In this configuration, the handcrafted and deep features are used separately for iPAD. The results of each iPAD system are scored to represent the probability of the input image belonging to either a real or presentation attack class. The two scores are combined by the weighted sum rule to make a final detection result as shown in Equation (12). In this equation, $S_{1}$ and $S_{2}$ are the decision scores of the PAD system based on only deep or only handcrafted image features, respectively. These scores are combined using two weight values of $w_{1}$ and $w_{2}$ whose sum is 1 as shown in Equation (13) to produce a final detection score *S*. In our experiment, we chose the optimal pair of $w_{1}$ and $w_{2}$ which produced the best classification accuracy of real and presentation attack on training dataset.

(12) $$S=w_{1}S_{1}+w_{2}S_{2}$$

(13) $$w_{1}+w_{2}=1$$

Using the SVM method, we classified the input images into either the real or presentation attack class. To evaluate the performance of our proposed iPAD method and to compare it with previous studies, we used the standard criteria, called average classification error rate (ACER), to measure the detection performance [2,46,47,48]. By definition, the ACER is a measurement of the average error rate of the attack presentation classification error rate (APCER) and the bona-fide presentation classification error rate (BPCER). In a PAD system, the APCER indicates the proportion of attack presentation images incorrectly classified as bona-fide presentation attack images, and BPCER indicates the proportion of bona-fide presentation attack images incorrectly classified as attack presentation images. The ACER was measured using Equation (14). Since the ACER indicates the error rate of a detection system, a lower value indicates better detection performance (small error). We used the training data to train the CNN model, PCA coefficients, and the SVM classifier. Consequently, the performance of the detection system (APCER, BPCER, and ACER) was measured using testing data. In experiments, we used the MATLAB environment for constructing and training the CNN model, image feature extraction, PCA, and SVM-based classification [49,50,51].

(14) $$ACER=\frac{APCER+BPCER}{2}$$

## 4. Experimental Results

### 4.1. Datasets

To evaluate the detection performance of our proposed iPAD method, we used two public datasets LivDet-Iris 2017-Warsaw [48] and Notre Dame Contact Lens Detection (NDCLD2015) [48,52]. For convenience, we refer to these datasets as Warsaw2017 and ND2015 in our study. Although there are other presentation attack iris image datasets such as IIITD-WVU, Clarkson [48], and PAVID [53], they were unavailable to us via internet request. In addition, the datasets we chose have been used in previous iPAD studies (LivDet-Iris 2017 competition [48]). The use of these datasets allowed us to compare the detection performance of our proposed method with those of previous studies.

The Warsaw2017 dataset contains 5168 real and 6845 presentation attack iris images obtained from 468 unique iris patterns with an image resolution of 640 × 480 pixels. This dataset was used in the LivDet-Iris 2017 iPAD competition and is the extended version of the two previous datasets of LivDet-Iris 2013 [54] and LivDet-Iris 2015 [52]. The presentation attack iris images in the Warsaw2017 dataset were collected by simulating a simple attack method by which the attackers use a printed sample of an iris pattern on paper to fool an iris recognition system during the image acquisition stage. A general statistical description of the Warsaw2017 dataset is given in the upper part of Table 4. Similar to the Warsaw2017 dataset, the ND2015 dataset was also used in the LivDet-Iris 2017 competition [48]. However, the presentation attack iris images in this dataset were simulated by iris patterns printed on a contact lens. Using this method, the presentation attack iris images look more like real ones than those of the Warsaw2017 dataset. The ND2015 dataset was first collected for the purpose of detecting whether a user used contact lenses [19]. This dataset was further used for detecting the presentation attack iris image in the LivDet-Iris 2017 competition because the fake iris images in this dataset simulate an attack method by which iris patterns are printed on the surface of a contact lens. In the lower part of Table 4, we show the general descriptions of the ND2015 dataset.

### 4.2. Detection Performance for Attack Method Based on Printed Samples

**Figure 10:**
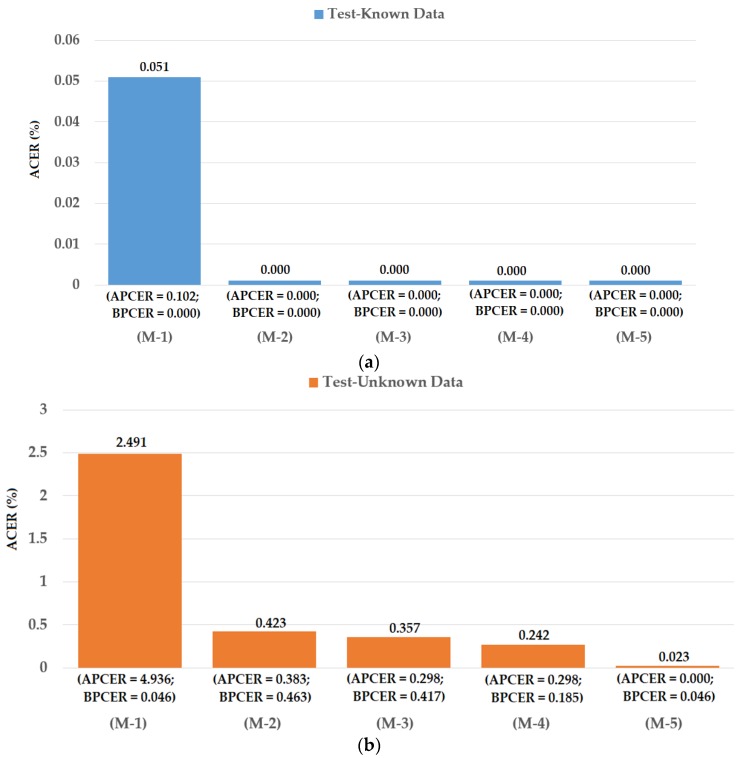
Detection errors of various iPAD methods using Warsaw2017 dataset: (**a**) Using Test-Known dataset; and (**b**) Using Test-Unknown dataset. Note: (M-1) Using CNN as Classifier; (M-2) Using CNN Features with PCA and Polynomial SVM Kernel; (M-3) Using MLBP Features with PCA and Polynomial SVM Kernel; (M-4) Using Feature Level Fusion with PCA and Polynomial SVM Kernel; and (M-5) Using Score Level Fusion with PCA and Polynomial SVM Kernel.

As our first experiment, we investigated the detection performance of our proposed iPAD method for the attack method based on printed paper samples. For this purpose, we used the Warsaw2017 dataset. In addition, we also measured the detection performances of iPAD systems that use only CNN method as classifier, CNN or MLBP features for comparison purposes. For evaluating the performance of an iPAD method, the Warsaw2017 dataset was preclassified into the three sub-datasets of training, test-known, and test-unknown. The training sub-dataset was used to construct the classification model, while the two testing sub-datasets were used for evaluating the performance of the trained model. The training and test-known sub-datasets were collected using the same capturing devices (Iris Guard AD 100), while the test-unknown dataset was collected using a different capturing device (a lab mate camera [48]). The use of the test-unknown dataset allowed us to evaluate the performance of the detection system for cross-sensor configuration. A detailed description of these training and testing sub-datasets is provided in Table 5. As shown in this table, we used 4513 images (1844 real and 2669 presentation attack images) for training. To test the detection model, 2990 images (974 real and 2016 presentation attack images) were used for the test-known dataset and 4510 images (2350 real and 2160 presentation attack image) were used for the test-unknown dataset. We generalized the training dataset by artificially making augmented images from original images to reduce the over-fitting tendency of the CNN method. In detail, we artificially made eight additional images from each original presentation attack iris image and an additional 14 images from each real iris image using shifting, cropping, and scaling method. This augmentation method has been also used in previous research [31]. Consequently, we increased the number of training images from 4513 to 51,681 images. The different number of artificial images for real and presentation attack was used because the number of original real iris images was much smaller than that of the presentation attack images. By using a different number of artificial images for each class, we made the number of images of each class similar in order to reduce over-fitting during the training process. A description of these sub-datasets and the corresponding augmented dataset are provided in Table 5. Data augmentation was performed for only the training data, and the testing data remained the same as the original. This approach was used to ensure a fair comparison of detection performance of our study with previous studies. Using the augmented train dataset, we performed the training procedure to train the CNN, PCA, and SVM models for the iPAD system. The experimental results on test datasets are given in Figure 10.

As shown in Figure 10, we obtained the best detection error of 0.000% using the test-known dataset for the iPAD systems using only CNN, MLBP, or hybrid features. There are two reasons for this result. First, the presentation attack iris images in the Warsaw2017 dataset were collected by recapturing the printed iris samples on paper. Therefore, the presentation attack iris images inherit many differences from real images such as broken textures and printing noise. In addition, as explained above, the test-known dataset was collected using the same capturing procedure and devices as the training dataset. Consequently, the characteristics of images in the training and testing datasets were very similar. Therefore, we obtained very good detection results using the test-known dataset. However, the situation was little changed using the test-unknown dataset. We obtained an error (ACER) of 0.423% using the iPAD method that used only CNN features with the polynomial kernel of the SVM method. The iPAD method that used only MLBP features produced an error of 0.357% using the polynomial kernel of the SVM method. Our proposed hybrid features iPAD method produced an error of 0.242% using the polynomial kernel of the SVM method. The iPAD system detection errors using the test-unknown dataset were higher than those using the test-known dataset because the test-unknown dataset was collected using different capturing devices than that of the test-known dataset. Consequently, it caused several differences in the characteristics of the images of the two datasets. From these results, we conclude that the hybrid features iPAD method outperformed the conventional CNN and MBLP image features by producing the lowest detection error.

**Figure 11:**
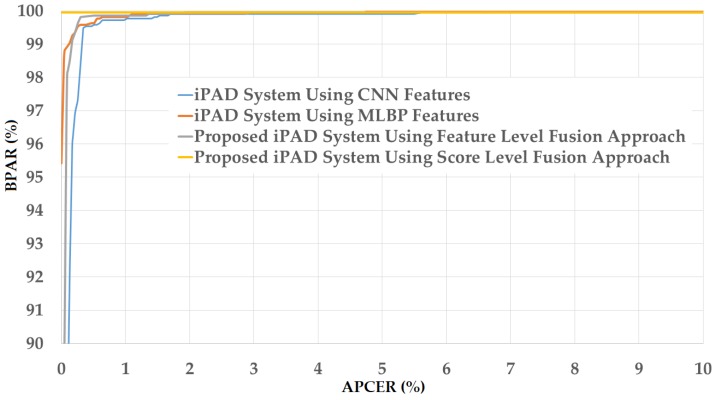
DET curves of iPAD systems based on use of CNN, MLBP, and hybrid image features (feature level fusion and score level fusion approach) using Warsaw2017 test-unknown dataset.

As a next experiment, we measured the detection errors produced by our proposed iPAD method based on score level fusion approach. Using the test-known dataset, we again obtained the same best detection error (ACER) of 0.000% as using the feature level fusion approach. For the test-unknown dataset, we obtained the best detection error of 0.023% using the combination rule of “polynomial-polynomial”. This error was much smaller than the error of 0.242% using the feature level fusion approach. Based on the experimental results, we can see that the combination of deep and handcrafted features was effective at enhancing the detection performance of the iPAD system. In addition, the score level fusion approach worked better than the feature level approach on the Warsaw2017 dataset. For demonstration, we show the detection error tradeoff (DET) curves of these experiments in Figure 11. In this figure, we drew the change of APCER according to the change in the bona-fide presentation acceptance rate (BPAR). The BPAR was calculated as 100—BPCER (%). Since the iPAD methods using only CNN, MLBP, or hybrid features perfectly detected presentation attack images for the test-known dataset, DET curves for these cases are meaningless. Therefore, we only show the DET curves of the four detection configurations using the test-unknown data in Figure 11. As shown in Figure 10 and Figure 11, we can see that the iPAD using combined features outperformed the iPAD system using CNN and MLBP features. In addition, the score level fusion outperformed the feature level fusion for the Warsaw2017 dataset. As shown at the beginning bars of Figure 10, we obtained detection errors of 0.051% and 2.491% using the CNN method as classifier (using the CNN method for directly classifying the real and presentation attack images) on the test-known and test-unknown datasets, respectively. These high detection errors indicate that our approach that uses the PCA for feature selection and SVM for classification is more efficient than the use of CNN method directly for iPAD. The reason is that the CNN network contains a huge number of parameters that make the CNN method usually faces with overfitting problem. As a result, redundant information can exist in extracted deep features, but it can be removed using PCA method.

### 4.3. Detection Performance for Attack Method Based on Contact Lens

As the second experiment in our study, we investigated the detection performance of our proposed iPAD for a presentation attack method based on contact lenses. For this purpose, we used the ND2015 dataset. As explained in Section 4.1, the ND2015 dataset was used in the LivDet-Iris 2017 iPAD competition. In this competition, the images in the ND2015 dataset were classified into training and testing datasets. They used a set of 600 real and 600 presentation attack images for a training dataset and a set of 900 real and 900 presentation attack images for a testing dataset. Similar to the Warsaw2017 dataset, two testing datasets were constructed including a test-known dataset (in which the presentation attack images were collected using the same contact lens manufacturer as that of the training dataset) and a test-unknown dataset (in which the presentation attack images were collected using contact lenses from a different manufacturer than that of the training dataset) [48]. However, the detailed information of how the images were divided into training and testing datasets was not available for us. In addition, the LivDet-Iris 2017 competition did not use the entire ND2015 dataset in its experiments. This approach can bias the detection results because only a small set of the dataset was used. Therefore, in our experiments using the ND2015 dataset, we considered three division methods for dividing the images into training and testing datasets.

**Figure 12:**
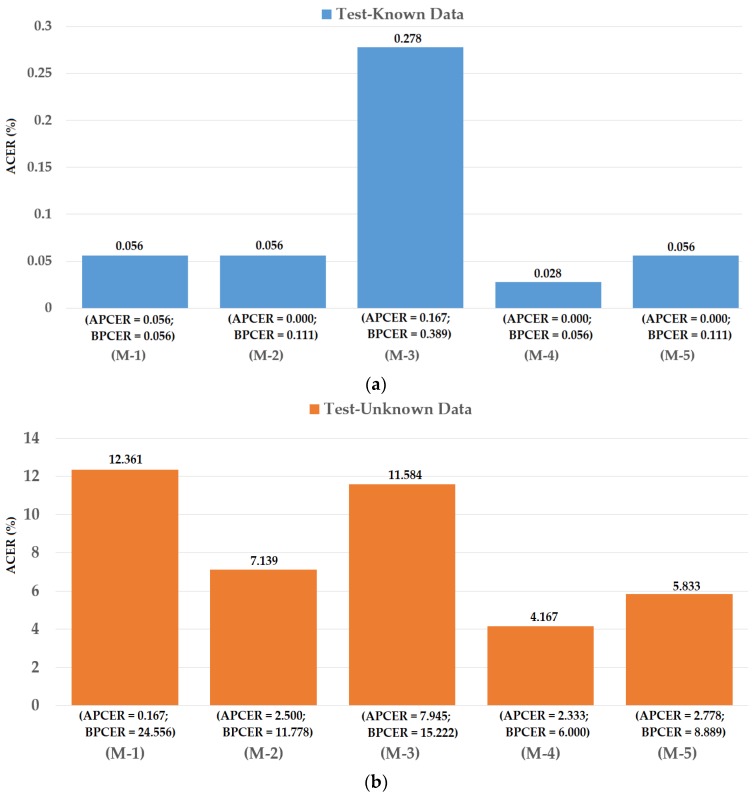
Detection errors of various iPAD methods using the first training-testing division method on ND2015 dataset: (**a**) Using Test-Known dataset; and (**b**) Using Test-Unknown dataset. Note: (M-1) Using CNN as Classifier; (M-2) Using CNN Features with PCA and Polynomial SVM Kernel; (M-3) Using MLBP Features with PCA and Polynomial SVM Kernel; (M-4) Using Feature Level Fusion with PCA and Polynomial SVM Kernel; and (M-5) Using Score Level Fusion with PCA and (Polynomial-Polynomial) SVM Kernels.

For the first division method, we performed the training and testing division approach similar to that of the previous study [48]. For this purpose, we divided images into training and testing datasets by randomly selecting images from the entire ND2015 dataset using the same criteria as the study by Yambay et al. [48]. The training dataset contained 600 real images (with no contacts, either soft or cosmetic) and 600 presentation attack images (with textured contact lenses manufactured by Ciba, UCL, and ClearLab) [48]. The test-known dataset contained 900 real and 900 presentation attack images and used contact lenses made by Ciba, UCL, and ClearLab (same as training data). The test-unknown dataset contained 900 real and 900 presentation attack images and used contact lenses made by Cooper and Johnson & Johnson [48]. The division procedure was performed by ensuring that there were no overlapped images in the three datasets. We iterated the above division procedure two times and performed experiments for measuring the detection performances because the information on dividing images into training and testing datasets in the study by Yambay et al. [48] was not available to us. As a result, the final detection performance was measured by averaging the detection results of the two iterated experiments. By using this division approach, we were able to fairly compare the detection performance of our proposed iPAD method with previous methods. In Table 6 we show the description of datasets used in the experiments, and in Figure 12 we show the experimental results.

**Figure 13:**
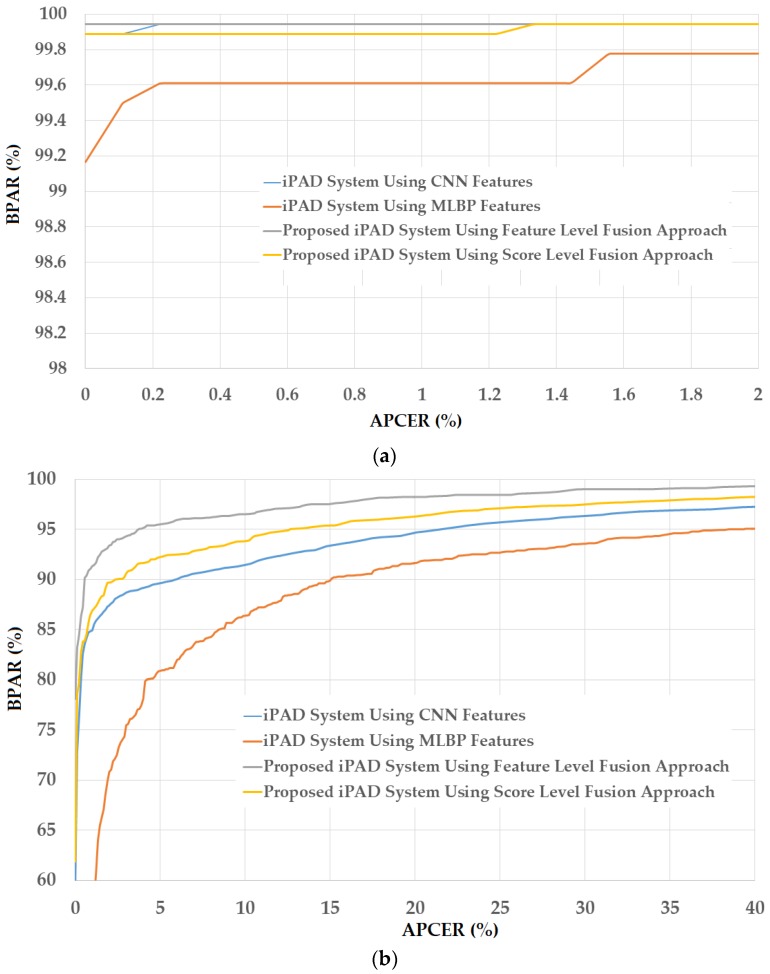
DET curves of iPAD systems based on use of CNN, MLBP, and hybrid image features (feature level fusion and score level fusion approach) using the first division method and ND2015 test-unknown dataset (**a**) DET curves of test-known dataset; and (**b**) DET curves of test-unknown dataset.

In Figure 12, we show the experimental results using our proposed method based on the feature level fusion approach. Using the test-known dataset, we obtained the best detection errors of 0.056%, 0.278% and 0.028% using the iPAD system based on only CNN feature, MLBP features, and hybrid features, respectively. Using the test-unknown dataset, these errors increased to 7.319%, 11.584%, and 4.167%. All these results were obtained using polynomial kernel of SVM method. In addition, we obtained an error of 0.056% for the case of using test-known data and 5.833% for the case of using test-unknown data using the score level fusion approach with ‘polynomial-polynomial’ combination rule. This detection error was higher than the error produced by the feature level fusion approach. However, this detection error was still lower than the detection errors produced by the iPAD systems using only CNN or MLBP features (ACERs of 7.139% and 11.584%, respectively). These results prove that our proposed iPAD method was effective at enhancing the detection performance of the iPAD system. In addition, the feature level fusion approach worked better than the score level fusion approach in our experiments using the ND2015 dataset. For demonstration purposes, we drew the DET curves of four system configurations using the test-known and test-unknown data in Figure 13. As observed from Figure 12 and Figure 13, we can see that our proposed method outperformed the conventional detection methods based on only CNN or MLBP features.

**Figure 14:**
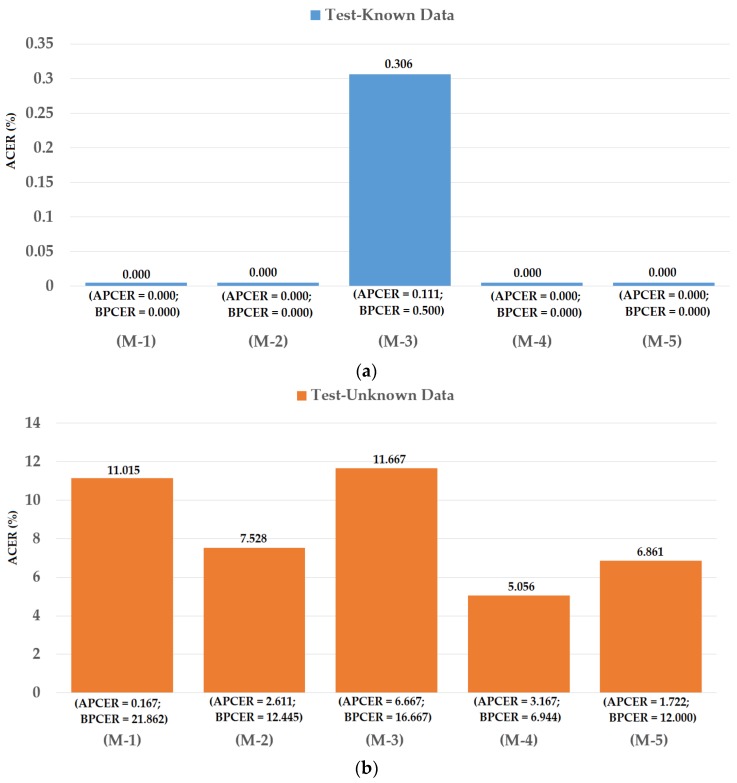
Detection errors of various iPAD methods using the second training-testing division method on ND2015 dataset: (**a**) Using Test-Known dataset; and (**b**) Using Test-Unknown dataset. Note: (M-1) Using CNN as Classifier; (M-2) Using CNN Features with PCA and Polynomial SVM Kernel; (M-3) Using MLBP Features with PCA and Polynomial SVM Kernel; (M-4) Using Feature Level Fusion with PCA and Polynomial SVM Kernel; and (M-5) Using Score Level Fusion with PCA and (Linear–Polynomial) SVM Kernels.

**Figure 15:**
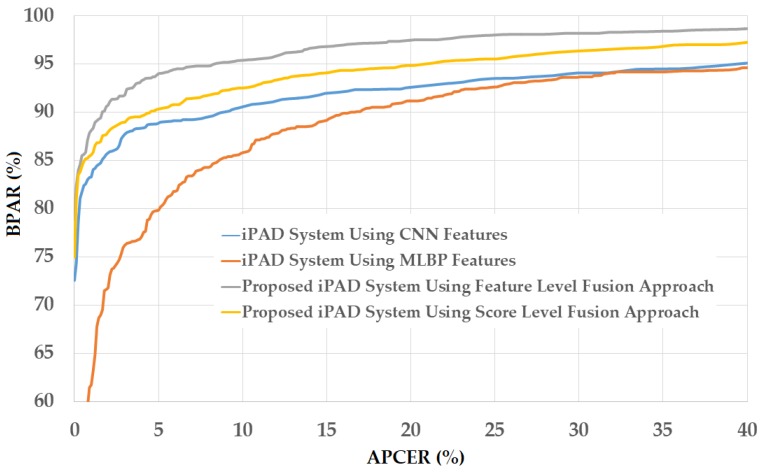
DET curves of iPAD systems based on use of CNN, MLBP, and hybrid image features (feature level fusion and score level fusion approach) using the second division method and ND2015 test-unknown dataset.

The first division method was performed using the same criteria as the division method used in LivDet-iris 2017 competition [48]. As a result, the real images were defined as the iris images without contact lens (with no contacts, either soft or cosmetic). However, there is a case in which users of iris recognition systems wear a soft (transparent) contact lens to protect their eyes or compensate their eye’s problem such as myopia or hyperopia. For this case, an iris recognition system should allow users using the system and the consequent iPAD method must consider an iris with soft contact lens as the real image ones. Based on this phenomenon, we re-performed the above experiment by considering the iris images with soft (transparent) contact lens as the real images ones. Similar to the first division method, we randomly selected 600 real images (with no contacts or with soft (transparent) contact) and 600 presentation attack images (with textured contact lenses manufactured by Ciba, UCL, and ClearLab) [48] for training dataset. By similar method, we selected the test-known and test-unknown datasets that contained 900 real and 900 presentation attack images. The number of images in training and testing datasets in this experiment is same as the above experiment and shown in Table 6. The detection results are provided in Figure 14. As shown in this figure, we obtained perfect detection performance (ACER of 0.000%) using the iPAD system based on CNN features or hybrid features on the test-known dataset. Using the MLBP features, the lowest average error of 0.306% was obtained. Similar to our experiments with the Warsaw2017 dataset, the detection error increased when we used the test-unknown dataset. We obtained the lowest detection errors (ACER) of 7.528% and 11.667% using the iPAD systems that use only CNN or only MLBP features, respectively. Using our proposed method based on the feature level fusion approach, the error was reduced to 5.056% using the polynomial kernel of the SVM method. Using the score level fusion approach, we obtained the lowest detection error of 6.861% using the “linear-polynomial” combination rule. This detection error was higher than the error produced by the feature level fusion approach (ACER of 5.056%). However, this detection error was still lower than the detection errors produced by the iPAD systems using only CNN or MLBP features (ACERs of 7.528% and 11.667%, respectively). These results prove that our proposed iPAD method was effective at enhancing the detection performance of the iPAD system. Furthermore, the feature level fusion approach worked better than the score level fusion approach in our experiments using the ND2015 dataset. For demonstration purposes, we drew the DET curves of four system configurations using the test-unknown data in Figure 15. We do not show the DET curves for the test-known dataset because we obtained perfect detection results using this dataset. As observed from Figure 14 and Figure 15, we can see that our proposed method outperformed the conventional detection methods based on only CNN or MLBP features.

**Figure 16:**
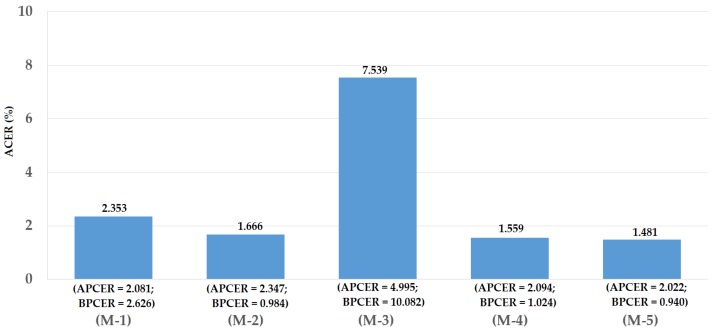
Detection errors of various iPAD methods using the third training-testing division method on ND2015 dataset. Note: (M-1) Using CNN as Classifier; (M-2) Using CNN Features with PCA and RBF SVM Kernel; (M-3) Using MLBP Features with PCA and RBF SVM Kernel; (M-4) Using Feature Level Fusion with PCA and RBF SVM Kernel; and (M-5) Using Score Level Fusion with PCA and (RBF–RBF) SVM Kernels.

**Figure 17:**
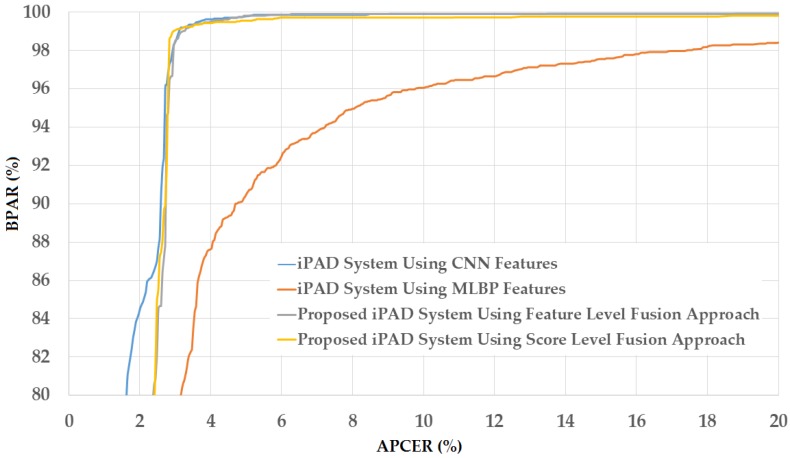
DET curves of iPAD methods based on use of CNN, MLBP, or hybrid image features (feature level fusion and score level fusion approach) using entire ND2015 dataset.

For the third division method, we used the entire ND2015 dataset for our experiments. For this purpose, we performed a two-fold cross-validation procedure to measure the detection accuracy of our proposed method. For the first fold, we divided the ND2015 dataset into training and testing datasets of which a half of ND2015 dataset was used for training and the other half for testing. The division was performed by ensuring that the images of the same individual only existed in either the training or the testing dataset. For the second fold, the training and testing datasets in the first fold were exchanged. By dividing the entire ND2015 dataset into training and testing datasets using this criterion, we were able to measure the detection accuracy using the entire dataset. In addition, this division approach divided images into the training and testing datasets without considering the difference in contact lens manufacturers. Therefore, we measured the detection accuracy in general. Based on this division method, we obtained the training and testing datasets as shown in Table 7. Similar to previous experiments, we performed data augmentation to generalize the training data. In Figure 16, we show the experimental results for this experiment. We obtained the best average detection accuracy (ACER) of 1.666% for the iPAD system using only CNN features and 7.539% for the iPAD system using only MLBP features. Both results were obtained using the RBF kernel of the SVM method. By using the feature level fusion approach, the detection error was reduced to 1.559%. The combination of two individual systems based on the score level fusion approach produced the lowest detection errors (ACER) of 1.481% using the RBF kernel in both subsystems. This detection error was lower than those produced by the two individual iPAD systems and the proposed iPAD system based on the feature level fusion approach. As shown in the experimental results in Figure 12, Figure 14 and Figure 16, our approach that uses the PCA for feature selection and SVM for classification on extracted CNN features outperformed the detection method that uses CNN as classifiers. For demonstration purposes, we show the DET curves of these experimental results in Figure 17. As demonstrated in the results, we can see that the proposed method was sufficient for iPAD. In addition, these detection accuracies were much better than those obtained in our previous experiment with the ND2015 dataset. The reason is that, in this experiment, we used a larger dataset for training the detection model, and we trained the detection model by merging all the possible cases of presentation attack images (without considering the known or unknown cases). This result suggests that we can obtain a much better detection accuracy when we collect enough data samples for training and perform testing with an attack method similar to that used in the training phase. However, this requirement is normally difficult to implement in real systems because various possible attack methods can be used in the testing phase that cannot be simulated in the training phase. To enhance the detection accuracy, we should simulate as many attack methods as possible for the training phase of the iPAD system.

### 4.4. Detection Performance for Attack Method Based on Both Printed Samples and Contact Lens

**Figure 18:**
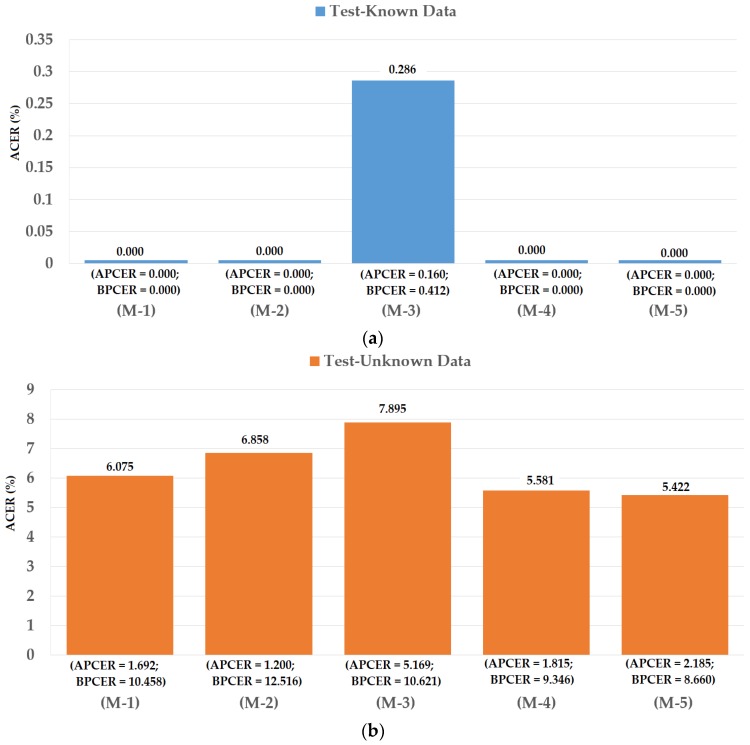
Detection errors of various iPAD methods using the fused dataset of Warsaw2017 and ND2015 datasets: (**a**) Using Test-Known dataset; and (**b**) Using Test-Unknown dataset. Note: (M-1) Using CNN as Classifier; (M-2) Using CNN Features with PCA and Polynomial SVM Kernel; (M-3) Using MLBP Features with PCA and Polynomial SVM Kernel; (M-4) Using Feature Level Fusion with PCA and Polynomial SVM Kernel; and (M-5) Using Score Level Fusion with PCA and (Linear–Polynomial) SVM Kernels.

As explained in Section 4.1, the presentation attack iris images in the Warsaw2017 and ND2015 datasets were collected by simulating two different attack methods, i.e., using printed samples (in the Warsaw2017 dataset) and contact lens (in the ND2015 dataset). The Warsaw2017 dataset was collected by recapturing the printed samples of real iris images. However, the ND2015 dataset was collected using a more complex attack method—the use of contact lenses. By performing experiments with each attack method, the detection system is only responsible for detecting presentation attack images for that given attack method. To make the detection accuracy robust for several kinds of attack methods, we performed experiments with a new dataset created by merging the Warsaw2017 and ND2015 datasets. By merging the two original datasets, the new dataset, named WARSAW-ND dataset in our study, contained real images captured using various cameras and capturing conditions and presentation attack images captured using two different attack methods as well as various capturing conditions. As a result, the WARSAW-ND dataset was more generalized than the Warsaw2017 and ND2015 datasets for iris presentation attack detection. For our experiment in this section, we combined the Warsaw2017 dataset (Table 5) and the ND2015 dataset (Table 6) to create the WARSAW-ND dataset shown in Table 8. For this experiment, we used the second division approach for dividing ND2015 dataset into training and testing datasets because it is reasonable for real applications. For the training dataset, we used 51,681 images from the Warsaw2017 dataset and 58,800 images from the ND2015 dataset. Using the same method, we created a test-known dataset containing 4790 images and a test-unknown dataset containing 6310 images for the experiment. Similar to the above experiments with the individual Warsaw2017 and ND2015 datasets, we performed experiments with the WARSAW-ND dataset using two system configurations based on feature level fusion and score level fusion. The experimental results are given in Figure 18.

**Figure 19:**
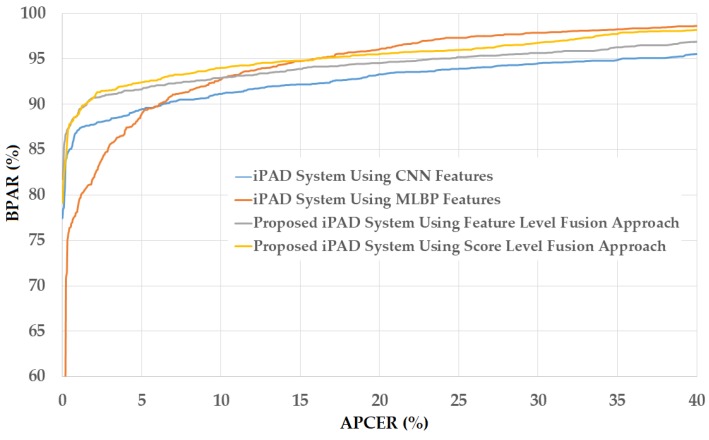
DET curves of iPAD systems based on use of CNN, MLBP, and hybrid image features (feature level fusion and score level fusion approach) using unknown data from a combination of ND2015 and Warsaw2017 datasets.

For the test-known dataset case, we obtained the best detection errors of 0.000%, 0.286%, and 0.000% using iPAD systems that use CNN features, MLBP features, and our proposed hybrid features, respectively. These results show that we obtained perfect detection using the test-known dataset. Similar to the explanations provided in Section 4.2 and Section 4.3, this result was caused by the fact that the test-known data were similar to the training data. However, the detection errors increased quickly for the test-unknown data case. We obtained the lowest detection errors of 6.858%, 7.895%, and 5.581% using the iPAD systems that use CNN features, MLBP features, and our proposed hybrid features, respectively. These detection results were much higher than those produced in the test-known data case. Using the score level fusion approach, the combination “linear-polynomial” rule produced the lowest detection errors with an ACER of 0.000% using test-known data and 5.422% using test-unknown data. These detection errors were equal for the test-known data case and lower for the test-unknown data case. However, the difference between the detection errors produced by the feature level fusion and score level fusion approaches was small (5.581% vs. 5.422%). From these results, we conclude that our proposed method is effective for enhancing the detection accuracy of iPAD systems whether they are based on the feature level fusion or the score level fusion approach. In addition, we again confirm that the iPAD system faces a significant problem with the unknown data because of the different capturing devices and contact lens manufacturers. For demonstration purposes, we drew the DET curves of the experimental results in Figure 19. We did not draw the curves for experiments using test-known data because we obtained perfect detection results with this data. This figure again confirms the efficiency of our proposed method over the individual methods based on only CNN or MLBP features.

### 4.5. Comparisons and Discussion

**Figure 20:**
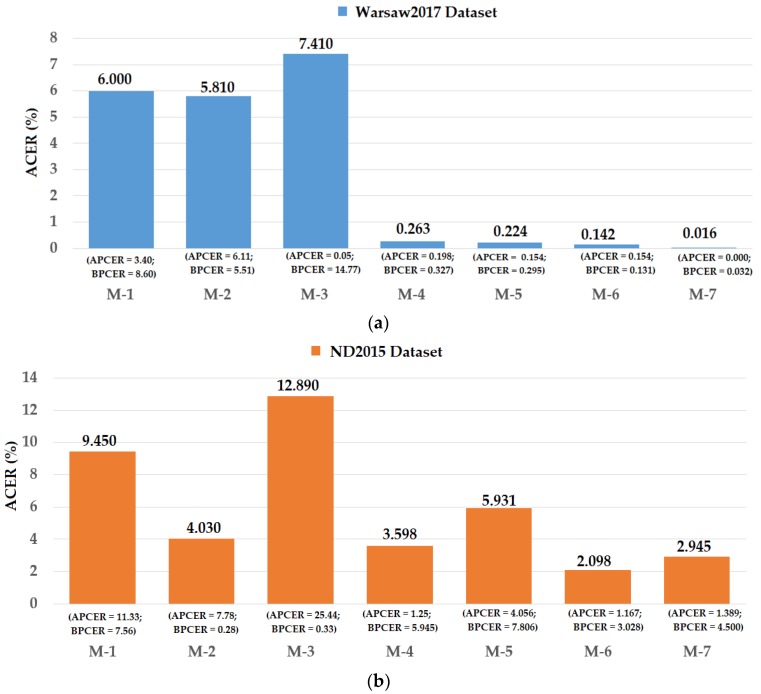
Comparison of detection error (ACER) between proposed method and previous methods using (**a**) Warsaw2017 and (**b**) ND2015 datasets. Note: (M-1) CASIA method [48]; (M-2) Anon1 method [48]; (M-3) UNINA method [48]; (M-4) CNN-based method [32]; (M-5) MLBP-based method [29]; (M-6) Proposed method based on feature level fusion; and (M-7) Proposed method based on score level fusion.

As explained in Section 4.1, Warsaw2017 and ND2015 datasets were used for the LivDet-Iris 2017 detection competition for iris recognition systems. In this competition, several detection methods were proposed by research groups, including CASIA, Anon1, and UNIA. To validate the detection performance of our proposed method, we performed a comparison of detection performances of our proposed method with those produced by previous methods used in the LivDet-Iris 2017 competition. The detailed comparison is shown in Figure 20. In this figure, the detection performances are given as the weighted average of detection errors of both the test-known and test-unknown datasets.

Using the Warsaw2017 dataset, the study by Yambay et al. [48] showed that the detection errors were about 6.00%, 5.81%, and 7.41% using the CASIA, Anon1, and UNINA methods, respectively. Using our proposed method, we reduced the detection error to 0.142% and 0.016% for the feature level fusion and score level fusion approaches, respectively. These detection errors were also lower than those of 0.263% and 0.224% produced by the iPAD systems using only CNN or MLBP features, respectively.

Using the ND2015 dataset, the work by Yambay et al. [48] obtained the best detection accuracy by using the Anon1 method with a reported detection error of 4.03%. As shown in our experimental results in Figure 20, our study obtained an error of 3.598% using the iPAD system using only CNN features. We obtained an average detection error of 5.931% using only MLBP features, which is still lower than the results obtained by the CASIA and UNINA methods [48]. Although the detection error produced by the iPAD system using only MLBP features was higher than that produced by the Anon1 method, the combination of the MLBP and CNN features using the feature level fusion approach produced an average error of 2.098%, which is much lower than the best detection error of 4.03% produced by a previous study [48]. In addition, although the detection error produced by our proposed method based on score level fusion was higher than that of the feature level fusion approach (ACER of 2.945%), this error was still lower than the best detection error reported by Yambay et al. [48]. From comparison with the very recent study on iPAD using the same datasets, we conclude that our proposed method outperforms previous studies and is an effective method for iPAD.

As shown in Figure 20, we obtained a very good detection result with the Warsaw2017 dataset. However, although the detection result for the ND2015 dataset was better than those produced by the previous study [48], it was still high compared to the results of the Warsaw dataset. The reason for this is that the Warsaw2017 dataset uses a very simple attack method and the consequent images in the Warsaw2017 dataset exhibit many noise components such as printing noise and broken texture that are easy to detect as shown in our experimental results in Section 4.2. However, by printing iris patterns on contact lenses for attack purposes, the iris patterns in the captured iris images in the ND2015 dataset display clearly without the additional negative components such as printing noise or broken texture features. In addition, a contact lens does not differentiate between real and presentation attack images on the non-iris regions such as the sclera, eyelid, eyelash, or skin regions. As a result, presentation attack images in the ND2015 dataset are more difficult to detect than those in the Warsaw2017 dataset.

In the CNN method of Yambay et al. [48], called spoofnet, the CNN network architecture with four convolution layers and one inception module was shallower than the CNN architecture of our study. In addition, we used the PCA method to select optimal image features and the SVM method to classify the input images based on extracted image features instead of using fully connected layers directly. As a result, our detection accuracy was higher than that of Yambay’s method. As shown in our experimental results, we also see that the cross-sensor or cross contact lens manufacturer is an important factor in an iPAD system. The use of a different capturing device for image acquisition or a different method to create a presentation attack iris image has a strong effect on a detection system by increasing the possibility of a successful attack on an iris recognition system.

## 5. Conclusions

In this study, we proposed a new PAD method for enhancing the security level of iris recognition systems. The main contribution of our proposed method is that we reduced the limitation of the deep learning-based method by using a combination of handcrafted image features and deep features. Although the deep learning-based method has proven to be effective for solving many computer vision problems, it still has several limitations such as over-fitting caused by the limited number of training data and the huge number of model parameters. As a result, the performance of the deep learning method is limited when applied to a problem which lacks training data. In our work, we used handcrafted image features designed by expert knowledge of PAD for an iris recognition system to extract the image features and extracted image features using the deep learning method. By combining the two kinds of image features, we enhanced the detection accuracy of a PAD system compared to previous studies. Using the popular Warsaw2017 and ND2015 public datasets, we showed that our proposed method outperformed previous methods by producing a much lower detection error rate as shown in Section 4. In addition, the polynomial kernel of SVM method works better than linear and RBF kernels in our experiments with Warsaw2017 and ND2015 datasets. We conclude that our proposed PAD method effectively enhances the security level of iris recognition systems.

## Figures and Tables

**Table 1:** Summary of previous studies on iPAD systems.

Category	Method	Strength	Weakness
Expert-knowledge-based feature extraction methods	- Uses local descriptors such as LBP, LPQ, and BSIF for detecting presentation attack image [18,19,20]; Eye movement information [21]; and color information [22].	- Easy to implement. - Do not require a large amount of training data.	- Detection accuracy varies according to dataset. - Cross-sensor problems.
Learning-based feature extraction methods	- Uses convolutional network to extract image features and neural network with SoftMax regression for classification [23]. - Uses CNN with structure and filter optimization [24].	- Good detection accuracy. - Image features are learned using a large amount of training data similar to that of human brain.	- More complex than use of handcrafted image features. - Over-fitting problem. - Requires large amount of real and presentation attack images to successfully train CNN network.

**Table 2:** Description of CNN architecture used for iPAD in our study.

Operation Group	Operation	Layer Name	Number of Filters	Filter Size	Stride Size	Padding Size	Output Size
Group_0 (G_0)	Input image	Input layer	n/a	n/a	n/a	n/a	224 × 224 × 3
Group 1 (G_1)	Convolution (2 times)	Convolution layer	64	3 × 3 × 3	1 × 1	1 × 1	224 × 224 × 64
		ReLU layer	n/a	n/a	n/a	n/a	224 × 224 × 64
	Pooling	Max pooling layer	1	2 × 2	2 × 2	0	112 × 112 × 64
Group_2 (G_2)	Convolution (2 times)	Convolution layer	128	3 × 3 × 64	1 × 1	1 × 1	112 × 112 × 128
		ReLU layer	n/a	n/a	n/a	n/a	112 × 112 × 128
	Pooling	Max pooling layer	1	2 × 2	2 × 2	0	56 × 56 × 128
Group_3 (G_3)	Convolution (4 times)	Convolution layer	256	3 × 3 × 128	1 × 1	1 × 1	56 × 56 × 256
		ReLU layer	n/a	n/a	n/a	n/a	56 × 56 × 256
	Pooling	Max pooling layer	1	2 × 2	2 × 2	0	28 × 28 × 256
Group_4 (G_4)	Convolution (4 times)	Convolution layer	512	3 × 3 × 256	1 × 1	1 × 1	28 × 28 × 512
		ReLU layer	n/a	n/a	n/a	n/a	28 × 28 × 512
	Pooling	Max pooling layer	1	2 × 2	2 × 2	0	14 × 14 × 512
Group_5 (G_5)	Convolution (4 times)	Convolution layer	512	3 × 3 × 512	1 × 1	1 × 1	14 × 14 × 512
		ReLU layer	n/a	n/a	n/a	n/a	14 × 14 × 512
	Pooling	Max pooling layer	1	2 × 2	2 × 2	0	7 × 7 × 512
Group_6 (G_6)	Inner Product	Fully connected layer	n/a	n/a	n/a	n/a	4096
		ReLU layer	n/a	n/a	n/a	n/a	4096
	Dropout	Dropout layer (dropout = 0.5)	n/a	n/a	n/a	n/a	4096
Group_7 (G_7)	Inner Product	Fully connected layer	n/a	n/a	n/a	n/a	4096
		ReLU layer	n/a	n/a	n/a	n/a	4096
	Dropout	Dropout layer (dropout = 0.5)	n/a	n/a	n/a	n/a	4096
Group_8 (G_8)	Inner Product	Output layer	n/a	n/a	n/a	n/a	2
	Softmax	Softmax layer	n/a	n/a	n/a	n/a	2
	Classification	Classification layer	n/a	n/a	n/a	n/a	2

**Table 3:** Parameters for training CNN models in our experiments.

Momentum	Mini-Batch Size	Initial Learning Rate	Learning Rate Drop Factor	Learning Rate Drop Period (Epochs)	Number of Epochs
0.90	32	0.001	0.1	3	9

**Table 4:** Description of Warsaw2017 and ND2015 datasets.

Dataset	Number of Real Images	Number of Attack Images	Total	Collection Method
Warsaw2017	5168	6845	12,013	Recaptured printed iris patterns on paper
ND2015	4875	2425	7300	Recaptured printed iris patterns on contact lens

**Table 5:** Description of training and testing data used with Warsaw2017 dataset.

Dataset	Training Dataset			Testing Dataset					
	Real Image	Attack Image	Total	Test-Known Dataset			Test-Unknown Dataset		
				Real Image	Attack Image	Total	Real Image	Attack Image	Total
Original dataset	1844	2669	4513	974	2016	2990	2350	2160	4510
Augmented dataset	27,660 (1844 × 15)	24,021 (2669 × 9)	51,681	974	2016	2990	2350	2160	4510

**Table 6:** Description of training and testing data used with ND2015 dataset.

Dataset	Training Dataset			Testing Dataset					
	Real Image	Attack Image	Total	Test-Known Dataset			Test-Unknown Dataset		
				Real Image	Attack Image	Total	Real Image	Attack Image	Total
Original ND2015 dataset	600	600	1200	900	900	1800	900	900	1800
Augmented dataset	29,400 (600 × 49)	29,400 (600 × 49)	58,800	900	900	1800	900	900	1800

**Table 7:** Description of training and testing data used for entire ND2015 dataset.

Dataset	Training Dataset			Testing Dataset		
	Real Image	Attack Image	Total	Real Image	Attack Image	Total
Original entire ND2015 (1st Fold)	2340	1068	3408	2535	1357	3892
Augmented dataset (1st Fold)	28,080 (2340 × 12)	26,700 (1068 × 25)	54,780	2535	1357	3892
Original entire ND2015 (2nd Fold)	2535	1357	3892	2340	1068	3408
Augmented dataset (2nd Fold)	30,420 (2535 × 12)	33,925 (1357 × 25)	64,345	2340	1068	3408

**Table 8:** Description of training and testing datasets of WARSAW-ND dataset.

Training Dataset			Testing Dataset					
Images from Warsaw2017 dataset	Images from ND2015 dataset	Total	Test-known dataset			Test-unknown dataset		
			Images from Warsaw2017 dataset	Images from ND2015 dataset	Total	Images from Warsaw2017 dataset	Images from ND2015 dataset	Total
51,681	58,800	110,481	2990	1800	4790	4510	1800	6310
